# Atmospheric particulate mercury at the urban and forest sites in central Poland

**DOI:** 10.1007/s11356-015-5476-5

**Published:** 2015-09-28

**Authors:** Patrycja Siudek, Marcin Frankowski, Jerzy Siepak

**Affiliations:** Department of Water and Soil Analysis, Faculty of Chemistry, Adam Mickiewicz University in Poznań, Umultowska 89b Street, 61-614 Poznań, Poland; Hipolit Cegielski State College of Higher Education in Gniezno, 38 ks. Kard. Stefana Wyszynskiego Street, 62-200 Gniezno, Poland

**Keywords:** Particulate mercury, Urban, Forest, Seasonal variation, Dry deposition, Poland

## Abstract

Particulate mercury concentrations were investigated during intensive field campaigns at the urban and forest sites in central Poland, between April 2013 and October 2014. For the first time, quantitative determination of total particulate mercury in coarse (PHg_2.2_) and fine (PHg_0.7_) aerosol samples was conducted in Poznań and Jeziory. The concentrations in urban fine and coarse aerosol fractions amounted to <MDL ± 77.1 pg m^−3^ and <MDL ± 604.9 pg m^−3^, respectively. Aerosol samples collected during the whole study period showed statistically significant differences for particulate mercury concentrations. A strong impact of meteorological conditions (wind velocity, air mass direction, air temperature, and precipitation amount) on particulate mercury concentrations was also observed. In particular, higher variation and concentration range of PHg_0.7_ and PHg_2.2_ was reported for wintertime measurements. An increase in atmospheric particulate mercury during the cold season in the study region indicated that coal combustion, i.e., residential and industrial heating, is the main contribution factor for the selected particle size modes. Coarse particulate Hg at the urban site during summer was mainly attributed to anthropogenic sources, with significant contribution from resuspension processes and long-range transport. The highest values of PHg_0.7_ and PHg_2.2_ were found during westerly and southerly wind events, reflecting local emission from highly polluted areas. The period from late fall to spring showed that advection from the southern part of Poland was the main factor responsible for elevated Hg concentrations in fine and coarse particles in the investigated region. Moreover, September 2013 could be given as an example of the influence of additional urban activities which occurred approx. 10 m from the sampling site—construction works connected with replacement of the road surface, asphalting, etc. The concentrations of particulate Hg (>600.0 pg m^−3^) were much higher than during the following months when any similar situation did not occur. Our investigations confirmed that Hg in urban aerosol samples was predominantly related to local industrial and commercial emissions, whereas the main source of Hg in particulate matter collected at the forest site was connected with regional anthropogenic processes. This paper provides the results of the first long-term measurements of size-fractionated particulate mercury conducted in central Poland, which could be an important insight into atmospheric Hg processes within such a scarcely investigated part of Europe.

## Introduction

Atmospheric chemistry of mercury is associated with a variety of natural and anthropogenic sources, among which local, regional, and global industrial and urban activities (coal combustion processes) play the most important role (Pacyna et al. 2010). Gaseous elemental mercury (GEM or Hg°) is the predominant form of Hg in the atmosphere (>95 % of total gaseous mercury (TGM), Ebinghaus et al. 1999), with residence time of about 1 year (Schroeder and Munthe 1998). The other Hg species which are present in the ambient air include oxidized and reactive gaseous mercury (GOM, RGM) and total particulate-phase species (TPM, PHg). They together constitute less than 5 % of TGM. Both these forms are less volatile, more water-soluble, and more chemically reactive than GEM and have much shorter atmospheric lifetime (i.e., minutes to weeks). Therefore, their vertical distribution in the troposphere is often limited to emission areas due to rapid removal processes via dry and wet deposition (Lindberg et al. 2007). However, depending on their chemical and photochemical transformations with oxidizing precursors as well as on the meteorological effects (turbulent mixing, inversion layer, entrainment of dry air), gaseous and particulate mercury usually vary significantly as a function of time and space.

Despite the significant decrease of total gaseous mercury registered at many background monitoring sites, urban environments are still regarded as hotspots in the regional and global budget of Hg (Xu et al. 2015). In recent years, field measurements from highly polluted regions in Asia (Fang et al. 2010; Fu et al. 2011; Zhu et al. 2014; Jen et al. 2014; Xu et al. 2015; Zhang et al. 2015), USA (Lynam and Keeler 2006; Rutter et al. 2009; Liu et al. 2010; Lynam et al. 2014) and Europe (Li et al. 2008), as well as multi-scale model analysis (Bieser et al. 2014; De Simone et al. 2014; Gencarelli et al. 2014) showed relatively high spatial and seasonal variability in concentrations of atmospheric particulate mercury. Furthermore, different source apportionment analyses (e.g., receptor model, hybrid chemical transport, positive matrix factorization) of speciated atmospheric mercury at most of those sites demonstrated a sharp increase in PHg as a result of anthropogenic emission from local point/non-point sources including refineries, iron/steel manufacturing sites, power and chemical plants, coal-fired utilities, and road traffic (Cheng et al. 2013; Li et al. 2008; Zhang et al. 2015). In addition, the influence of high-temperature processes associated with residential and industrial heating during cold season was emphasized in the abovementioned works. For example, extremely high values of total particulate Hg concentrations (8407 pg m^−3^) were observed in the city of Guiyang, one of the most polluted areas in China, which reflected a large contribution of urban activities, such as residential coal burning and smelting (Fu et al. 2011).

The long-term Hg measurements in central and eastern Europe are still limited. So far, the observations of speciated atmospheric mercury (wet and dry deposition) in Poland have been conducted only in the urbanized coastal zone of the southern Baltic (Beldowska et al. 2012; Siudek et al. 2015), in the Upper Silesia region (Pyta et al. 2009), and at the single rural site in southern Poland (Zielonka et al. 2005). Siudek et al. (2011) studied the variability of total particulate mercury (TPM) in Gdynia (northern Poland) over the 1-year study period in 2008–2009, using the Principal Component Analysis method, and found large wintertime fluctuations of Hg both in fine and coarse aerosol fractions, with the mean value of 4.1 ± 6.7 pg m^−3^ and 35.5 ± 28.5 pg m^−3^, for TPM_0.7_ and TPM_2.2_, respectively. These investigations provided a significant insight into pollution sources, atmospheric processes, seasonal patterns, and various factors controlling the concentrations of particulate mercury at urban sites. However, they did not provide data of reactive gaseous species (RGM). Although both the northern and southern parts of Poland have started atmospheric mercury field programs, there are still no reliable data of particle-bound mercury concentrations and dry deposition fluxes from industrialized and urbanized central regions such as Wielkopolska district. In this paper, we present the first long-term measurements of particulate mercury in the urban and forest sites in central Poland. The main purposes of this study were as follows: (1) examine seasonal variation of PHg_0.7_ and PHg_2.2_ within the two-point study domain, (2) identify main factors (chemical and meteorological) that affect particulate-phase mercury concentrations, (3) determine types of emission sources and factors responsible for seasonal variability in particulate-phase mercury, including urban and forest areas, and (4) estimate dry deposition fluxes of particulate mercury. We also compared our results with other urban/industrial and remote/forest areas.

## Materials and methods

### Study area

**Fig. 1:**
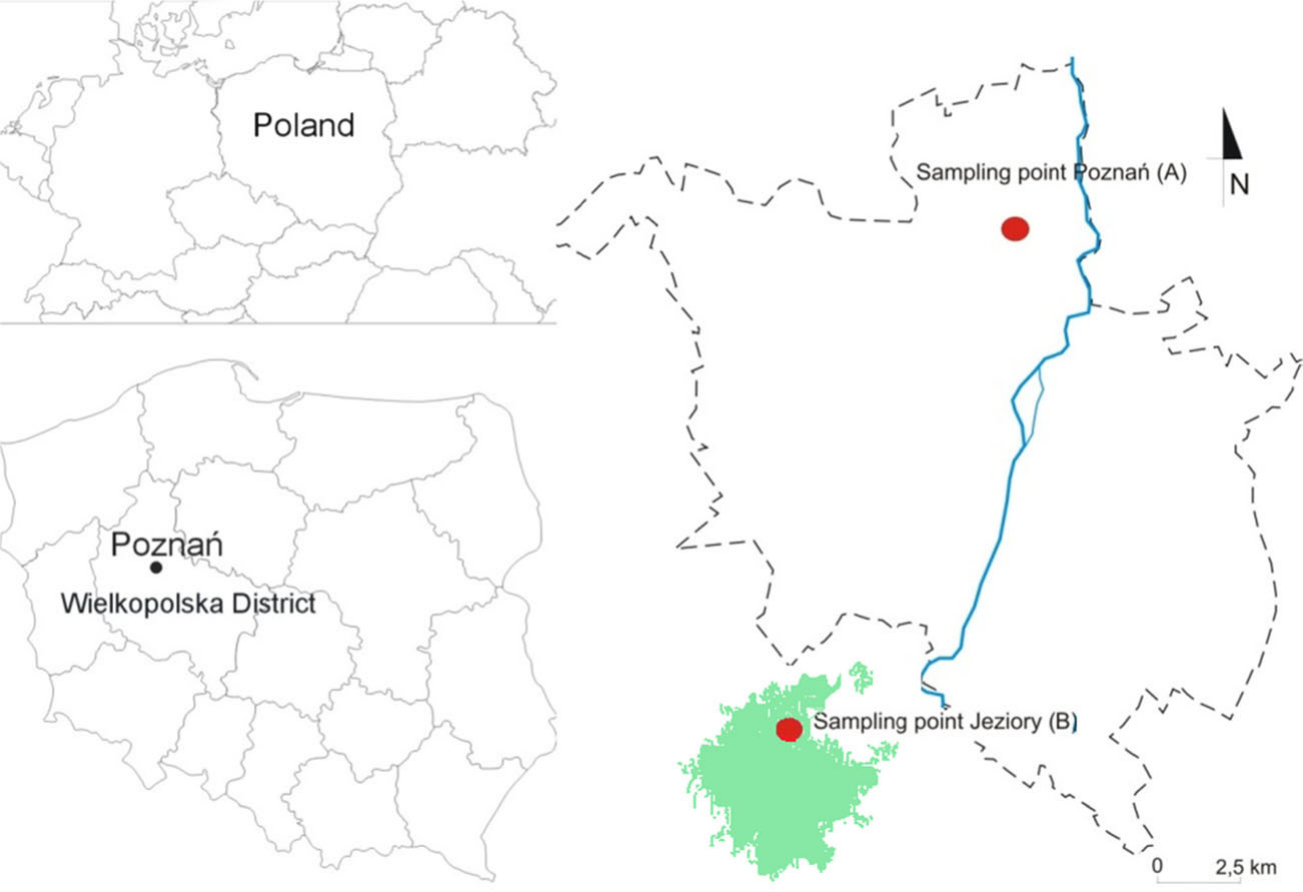
Location of both sampling sites: Poznań (**a**) and Jeziory (**b**) in Wielkopolska District, central Poland. The mixed forest has been indicated as *green area*

Aerosol samples for mercury analysis were collected at two sites in Wielkopolska district (Poland) during the 1.5-year study period between April 2013 and October 2014. Poznań is the largest city in this region, with the population of about 700,000 (Fig. 1). The first sampling site was located in the Botanic Garden of Adam Mickiewicz University (52° 42′ N, 16° 88′ E, Fig. 1), approx. 2 km northwest from the city center of Poznań. The airport Poznań Ławica is located approx. 4 km west of this station. About 10 km northeast of the sampling site, there is a large coal-fired power plant—Karolin CFPP. In addition, within a radius of 30 km of Poznań, several major sources of Hg are located, i.e., dumping grounds for municipal wastes, low-capacity domestic heating units, sewage treatment plants, cement factories, industrial units producing metal and paints, smelters, waste incinerators, different manufactories, heavy traffic, and agricultural activities.

The second site was located in the Ecological Station of Adam Mickiewicz University in Jeziory (Fig. 1). This station is situated about 30 km southwest of Poznań Agglomeration, in the protected woodland area of the Wielkopolski National Park. Any particular local anthropogenic urban or industrial activities are not closer than within approx. 25 km of the site, and traffic emission is relatively low (medium-traffic road is about 4 km away). Hence, this sampling station can be regarded as a regional background site for atmospheric mercury measurements.

### Sampling site and program

In both locations, particulate mercury samples were collected using a low-volume sampler. The sampling system included oil-free vacuum pump, air flow gauge inside the sampling box, filter holder, and acid-cleaned open-faced Teflon filter pack installed at the 2-m sampling tower. Filters were loaded in a cascade (downward facing) and closed in a Teflon case.

The filter-based sampling system was used for the collection of particle-phase Hg samples onto two types of 47-mm filters: quartz-fiber filters (with pore size of 2.2 μm, for coarse particles collection, operationally defined as PHg_2.2_ or Hg_coarse_) and glass-fiber filters (with pore size of 0.7 μm, for fine particles collection, operationally defined as PHg_0.7_ or Hg_fine_). The sampler was operated at the average air flow rate of 30 L min^−1^. Prior to sampling, quartz filters were pre-combusted for 5 h at 500 °C in a muffle furnace in order to remove all organic compounds. During the whole study period, a total number of 326 samples were collected at both sites using the same instrumentation; however, the aspiration time was different. At the POZ sampling site (urban area), the filter pack was prepared for a 24-h sampling period (typical aspiration cycle: 7 a.m. to 7 a.m. of the next sampling day), except for non-working days when a 72-h sampling procedure was applied. At the second station, in Jeziory, field measurements were carried out continuously for about a week. Such an approach was implemented in order to collect a measurable amount of particulate mercury as well as to maintain maximum efficiency of the sampling system and to improve the sensitivity of Hg detection. Different sampling durations at two sites could lead to some positive (RGM sorption or reaction with deposited solid phase on the filter) or negative (volatilization of unknown part of semi-volatile species through gas-particle conversion) artifacts, especially at the forest site. A detailed description of errors associated with long sampling time can be found elsewhere (Lynam and Keeler 2005). After sampling, filter samples were sealed in separated polyethylene zipped bags and stored at −18 °C until the main analysis.

The use of undenuded quartz filters made it impossible to estimate what percentage of gaseous Hg species (i.e., RGM) was retained during the aspiration period, due to lack of an attached KCl-coated annular denuder to trap gas-phase Hg° and Hg^2+^ from the sampled airstream. Therefore, based on the results from other observations, i.e., Lynam and Keeler (2002, 2005) and Landis et al. (2002), we can only speculate about possible uncertainties estimated during the particulate mercury sampling with a filter pack containing quartz filters. Lynam and Keeler (2005) reported a large spectrum of errors while determining particulate mercury concentrations in 10-, 14- and 24-h duration samples collected onto quartz filters through the use of (i) KCl denuder (for Hg^2+^ removal), (ii) KCl and KI denuders (for O_3_ and Hg^2+^ removal), and (iii) without a denuder system. For example, they observed larger amounts of particulate mercury on denuded filters compared to undenuded ones for the majority of sampling days (Lynam and Keeler 2005). It was found that the maximum difference between denuded and undenuded filters was 33 pg m^−3^. Interestingly, results from the experiments with KCl-coated denuders suggested that homogeneous or heterogeneous chemical reactions may take place inside a denuder during sampling, causing statistically significant net production of Hg. The authors highlighted that RGM production may also occur in KCl denuders (Lynam and Keeler 2005). Moreover, comparison tests indicated that ozone can be scrubbed from KCl denuders and the efficiency of this mechanism decreases with the increase in ozone concentration (Lynam and Keeler 2005). All the artifacts mentioned above are mostly associated with higher photochemical activity and higher levels of oxidants in the atmosphere. The filter-based sampling approach is still one of conventional methods in particulate mercury investigations (Lu et al. 1998) and has been applied by other groups of aerosol scientists in Poland during Hg measurement campaigns (Zielonka et al. 2005; Pyta et al. 2009; Siudek et al. 2011; Beldowska et al. 2012). In addition, there are some contradictory results that showed significantly higher amounts of particulate mercury collected on undenuded quartz filters as compared to those collected downstream of KCl-coated annular denuders (Lynam and Keeler 2002; Landis et al. 2002). In such cases, the observed artifacts were caused by a presence of Hg^2+^ which was absorbed by particulate matter.

It the present study, we did not perform any inter-comparison experiments to quantify positive or negative artifacts between the conventional sampling methods (filter pack with quartz filters) and the methods with the use of KCl-coated annular denuder (for reactive gaseous mercury removal). Therefore, while estimating total particle-bound Hg, we did not discuss any uncertainties arisen from the lack of RGM separation by a KCl-coated denuder. The results of particulate-phase mercury concentrations were obtained from both types of filters, representing the total particle-phase mercury (Hg_p_ = PHg_0.7_ + PHg_2.2_).

### Analysis of Hg in particulate matter

Mercury concentrations were quantitatively determined using a cold-vapor atomic fluorescence spectrometry (CVAFS, PSA 10.025 Millennium Merlin, UK) following EPA method 1631E (US EPA 2002). The instrument was optimized using five standard solutions (5, 10, 15, 20, and 50 ng L^−1^ of Hg, *R*^2^ > 0.999), prepared from a HgNO_3_ stock solution, to ensure stable conditions over the whole analytical procedure. An average recovery level of Hg_p_ was 98.3 ± 1.6 %, and the method precision (given as relative standard deviation) was found to be below 8 % (*n* = 6). The detection limit for the Hg_p_ analysis, calculated as three times the standard deviation of a set of 10 analytical blanks, was 1.7 pg m^−3^. Prior to the analysis, filter samples were acid-digested (10 mL of 60 % HNO_3_) using the microwave digestion system (MARSXpress), in acid-cleaned Teflon vessels. The analytical QC/QA was performed using a series of blanks and calibration curves in order to evaluate any loss of Hg during the experiments. The series of field (the pre-loaded filter pack with quartz and glass-fiber filters, connected to the sampling system but without air pumping) and procedural blanks were analyzed in the same manner as environmental samples. Blank values corresponded, on average, to 1.8 % of a sample value (*n* = 6). The value of the field blank was subtracted from total Hg_p_ concentration measured in each sample. The collection, handling, transport, and storage did not introduce any significant artifacts.

### Backward trajectories and meteorological data

The HYSPLIT model (Draxler and Rolph 2003, NOAA Air Resources Laboratory, Silver Spring, MD, USA) was used to study air masses passing over the study domain and to identify potential sources that emerged during the long-range transport of air parcels towards the sampling location. The input parameters were as follows: meteorological database—Global Data Assimilation System (GDAS, spatial resolution 1°), starting heights above ground level—500/1000/1500 m, trajectory duration—96-h, and the vertical motion based on model vertical velocity. The backward trajectories (BTs) of air parcels were generated at 6-h intervals (at 0:00 am, 6:00 am, 12:00 pm, and 6:00 pm) for each event.

The meteorological data considered in this paper included air temperature, relative humidity, atmospheric pressure, wind speed, and direction. All data were registered automatically using a meteo-station at both sampling sites. In general, average air temperature, pressure, and relative humidity in Poznań ranged between −5.7 and 23.6 °C, 1000 and 1008 hPa, 76 and 95 %, respectively. At the Jeziory station, in the period between April 2013 and October 2014, the values of air temperature were between −10.0 and 24.8 °C, relative humidity was 66 %, while the monthly mean pressure varied between 1000.7 and 1008.0 hPa.

Statistical analyses were performed through the use of Statistica v.10.0 software. The non-parametric Kruskal-Wallis test was applied to determine differences of PHg_0.7_ and PHg_2.2_ in relation to all seasons, i.e., spring (III–V), summer (VI–VIII), fall (IX–XI), and winter (XII–II) and sites. Data were analyzed for the normality, and the outlier/extreme values were determined. For all tests, the *p* value of <0.05 was considered as statistically significant.

A theoretical model was applied to calculate dry Hg deposition fluxes (F_d_ in μg m^−2^ period^−1^). Similar approach was previously used by Fang et al. (2010) and Wan et al. (2009).

## Results and discussion

### Particulate mercury in central Poland: urban vs. forest site

Results from a 1.5-year study period in Poznań and Jeziory represent the first insight into the atmospheric chemistry of particulate mercury over a polluted region in central Poland. During these field measurements, mean concentrations of size-fractionated particulate mercury, operationally defined as fine (Hg_fine_) and coarse modes (Hg_coarse_), demonstrated statistically significant differences at the sampling sites. This significance was confirmed by the Kruskal-Wallis test (*p* < 0.05). Specifically, at the POZ site, the median value of Hg concentration in coarse particles was four times higher compared to Hg in fine particles (Table 1).

**Table 1:** Statistical analysis of particulate mercury concentration (pg m^−3^) determined in fine and coarse aerosol samples at the sampling sites in Poznań (urban) and Jeziory (forest), between April 2013 and October 2014. The MDL is the amount of particulate mercury below the method detection limit

	Poznań		Jeziory	
	Hg_fine_	Hg_coarse_	Hg_fine_	Hg_coarse_
Mean	7.3	22.6	2.4	20.8
SD	9.1	45.3	2.8	21.6
Median	4.0	16.4	1.6	13.9
Q_1_–Q_3_	1.5–10.0	8.3–31.3	0.5–3.1	6.3–30.4
Range	<MDL–77.1	<MDL–604.9	<MDL–16.1	<MDL–142.5
5–95 % quartile	0.3–22.8	2.3–76.5	0.2–8.3	1.4–62.8
*n*	226	226	100	100

At the urban sampling site, mercury concentrations in fine particles ranged from <MDL to 77.1 pg m^−3^, with the average of 7.3 ± 9.1 pg m^−3^. The Hg concentration in coarse particles was significantly higher at this site (mean value ± SD 22.6 ± 45.3 pg m^−3^). The 75 % of Hg measurements obtained for coarse particles in Poznań had concentrations up to 31.3 pg m^−3^, whereas the upper quartile of Hg in fine particles corresponded to the values below 10.0 pg m^−3^ (Table 1). At the second site, in Jeziory, the mean ± SD of Hg concentration in coarse and fine particles was 20.8 ± 21.6 pg m^−3^ and 2.4 ± 2.8 pg m^−3^, respectively. In addition, 90 % of Hg_fine_ values were within the range of 0.2–8.3 pg m^−3^ at this site.

### Particulate mercury from other ground-based observations

The strong contrast between concentrations of total particulate mercury over urban/industrial and rural/remote sites in Europe, USA, Canada, and Asia has been presented in Table 2.

**Table 2:** Comparison of particulate mercury measurements from various worldwide sites. PHg concentration values are in pg m^−3^, sampling sites are labeled as follows: (*C*) coastal, (*R*) rural, (*Re*) remote, (*U*) urban, and (*F*) forest

Site	Site type	Season	PHg	Reference
Poznań, Poland	U	April 2013–October 2014	Coarse, <MDL–604.9@fine, <MDL–77.1	This study
Gdynia, Poland	C/U	April 2008–April 2009	Coarse, 0.3–151.5@fine, 0.2–39.9	Siudek et al. (2011)
Gdynia, Poland	U	December 2007–December 2008	2–142	Beldowska et al. (2012)
Detroit, USA	U	2004	1.0–1345.2	Liu et al. (2010)
Femman, Sweden	U	2005	3.89–20.26	Li et al. (2008)
Beijing, China	U	2003–2004	180–3510	Wang et al. (2006)
Toronto, Canada	U	December 2003–November 2004	21.5	Song et al. (2009)
Mexico City, Mexico	U	March 2006	187 ± 300	Rutter et al. (2009)
Xiamen, China	U	March 2012–February 2013	174.4	Xu et al. (2015)
Guiyang, China	U	August–December 2009	0–8407	Fu et al. (2011)
Seoul, Korea	U	2006	23.9 ± 42.6	Seo et al. (2012)
Toronto, Canada	U	December 2003–November 2004	14.2–39.2	Zhang et al. (2012)
Changchun, China	U	1999–2000	276	Fang et al. (2004)
Nanjing, China	U	June 2011–February 2012	320–2040	Zhu et al. (2014)
San Francisco Bay Area, USA	U	2008	80.8 ± 283	Rothenberg et al. (2010)
Jeziory, Poland	F	April 2013–October 2014	Coarse, 20.8 fine, 2.4	This study
Waldhof, Germany	R	2009–2011	<0.4–262	Weigelt et al. (2013)
Dexter, USA	R	2004	1.0–90.56	Liu et al. (2010)
St. Anicet, Canada	R	2003	26 ± 54	Poissant et al. (2005)
Alert, Canada	Re	2002–2011	41.3	Steffen et al. (2014)
San Francisco Bay Area, USA	R	2008	7.99 ± 6.74	Rothenberg et al. (2010)

The mean concentration of total particulate mercury in coarse aerosol samples from Poznań was higher than values reported for some urban locations. For instance, Li et al. (2008) noted that the mean 24-h particulate-phase mercury concentration over the polluted area in Göteborg, Sweden did not exceed 12.5 ± 16.4 pg m^−3^. Similar results were also obtained from measurements in Toronto (mean 18.0 pg m^−3^, Song et al. 2009) and a slightly higher mean Hg_p_ concentration value was found in Seoul (26.3 ± 42.6 pg m^−3^, Seo et al. 2012).

The average total Hg_p_ concentration in Poznań was about six times lower than that observed at the urban site in Mexico City, Mexico (Rutter et al. 2009). Some other measurements from highly polluted megacities in Asia, e.g., Guiyang (Fu et al. 2011), Beijing (Wang et al. 2006), Changchun (Fang et al. 2004), Nanjing (Zhu et al. 2014), or USA, e.g., Detroit (Liu et al. 2010) and San Francisco Bay Area (Rothenberg et al. 2010), exhibited elevated levels and significantly higher variability of total particulate mercury as compared to the city of Poznań, which suggests a large contribution from different anthropogenic sources to the total Hg_p_. Results from the abovementioned studies showed that the particulate fraction of atmospheric mercury was predominantly affected by local industrial emission (power plants, cement production, non-ferrous refinery, metallurgical processes, waste incinerator, steel industry, rubber and aluminum plants, glass factory) and other non-point Hg sources. For example, at the sub-urban site in Xiamen, China, the Hg_p_ concentrations varied between <MDL and 2930 pg m^−3^, with the mean value of 174.4 pg m^−3^, and were mainly associated with industrial and vehicle emissions (Xu et al. 2015). Similarly, Xiu et al. (2009) measured significantly higher Hg concentrations in traffic-originated particulate matter at the measurement site near a road (20 · 10^3^ vehicles hr^−1^ in rush hour) in Shanghai, China, suggesting a large contribution from tailpipe exhaust, wear dust from tires or brake linings, and the resuspension of road dust. Levels of Hg_p_ measured at our urban site were lower than those determined at several sites in the vicinity of a semiconductor manufacturing complex in Taiwan, with higher Hg_p_ (0.26 ng m^−3^) observed in summer (Jen et al. 2014).

As shown in Table 2, particulate matter samples collected at the Jeziory site had the same mean values of Hg concentrations as those reported by Poissant et al. (2005), however, slightly higher as compared to rural sites in San Francisco Bay Area, North America (Rothenberg et al. 2010) or in Dexter (Liu et al. 2010). Interestingly, the Hg_p_ concentrations in Jeziory were on average two times lower in comparison with the remote polar site in Alert, Canada, where a significant increase of Hg_p_ in the ambient air was observed under specific conditions during the AMDE, i.e., very low temperature (*T* = −24.8 °C) and relatively high particle loadings (Steffen et al. 2014). Such conditions favor the partitioning of oxidized mercury from RGM to Hg_p_. The range of total particulate-bound mercury (≤2.5 μm) observed during a 3-year measurement cycle at a rural background site in Germany (Weigelt et al. 2013) was higher than the values obtained at the forest site in this study.

In the present study, Hg_coarse_ was predominant at both sites and constituted, on average, 76 and 90 % of Hg_p_ in Poznań and Jeziory, respectively. This was slightly lower as compared to the data reported for Gdynia by Beldowska et al. (2012). They found the Hg_coarse_/Hg_p_ ratio to be 0.93. This result can be explained by much higher contribution of Hg adsorbed onto sea-salt aerosols, which were directly emitted from bursting bubbles and breaking waves (Beldowska et al. 2012). During our 1.5-year study, the urban/forest ratio of mean Hg_coarse_ and Hg_fine_ concentrations was calculated to be 1.1 and 3.0, respectively, indicating a significant role of fine particles in atmospheric mercury transformations in Poznań. Recent studies by Liu et al. (2010) showed a twofold higher median Hg_p_ concentration at the urban site (Detroit) than at the rural site (Dexter), indicating a significant impact of urban/industrial areas on less polluted surrounding regions.

### Seasonal variations in the concentration of particulate mercury

**Fig. 2:**
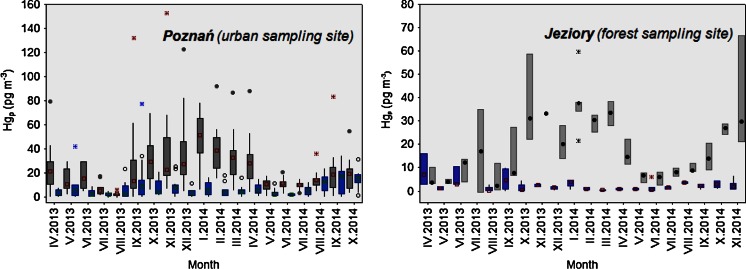
Seasonal variation of monthly particulate mercury concentrations (pg m^−3^) in Poznań (*left*) and Jeziory (*right*), between April 2013 and October 2014. The Hg_coarse_ is indicated with *gray* box-whisker diagram and Hg_fine_ with *blue*. The extreme values measured in Poznań (604.9 pg m^−3^, September 2013) and Jeziory (142.5 pg m^−3^, September 2013) have been excluded

Figure 2 shows monthly average concentrations of Hg_p_ at both examined sites in central Poland, between April 2013 and October 2014. The seasonal variability of Hg_p_ concentration in coarse particles at both locations was quite similar. However, Hg_coarse_ values determined for Poznań were significantly higher (Kruskal-Wallis test, *p* = 0.001). Specifically, the period of elevated mercury concentrations in coarse particles corresponded to the fall season of 2013 (mean 46.1 pg m^−3^) and the winter season of 2013/2014 (mean 42.3 pg m^−3^), whereas the lowest values of Hg_coarse_ were found in summer 2013 (mean 8.3 pg m^−3^). The same seasonal trend was observed for fine particulate Hg in Poznań, with mean Hg_fine_ concentrations of 7.3 and 5.2 pg m^−3^, respectively in winter and summer (Fig. 2). These differences were statistically significant (*p* < 0.05). In 2014, the highest mean concentration of Hg in coarse particles was observed in January at the urban site (48.1 pg m^−3^), while the monthly minimum was found in July (9.3 pg m^−3^). Moreover, the results from this site showed clear seasonal changes. The independent means *t* test (*t* = 6.301758, df = 63, *p* < 0.05) confirmed that differences in mean concentrations of Hg_coarse_ between warm and cold seasons of 2014 were statistically significant. These inter-seasonal variations in the atmospheric chemistry of Hg, registered at the POZ sampling site, could be partly explained by substantial changes in meteorological conditions, i.e., differences in air temperature and humidity, variable wind speed and direction, turbulent diffusion rates, height of the mixing layer, and strength of various emission sources. For instance, higher concentrations of Hg_coarse_ in the period between October 2013 and February 2014 corresponded mostly to lower air temperatures. The negative correlation (*R* = 0.63) between Hg_coarse_ (>30.0 pg m^−3^) and ambient air temperature (*T*_air_ range −9.4 to −1.1 °C) was found in February 2014. It was previously observed that low temperature is an important factor in atmospheric Hg transformations, and it enhances gas-to-particle conversion through the condensation and coagulation of combustion compounds onto aerosol surface (Kim et al. 2012).

In Poznań, the combustion of fossil fuels for domestic heating and power plants seems to be a key process responsible for notable increases in the concentration of Hg in particulate matter. The intensive hard coal combustion in low-capacity domestic heating units (DHU) during heating season was previously identified as a main contributor of speciated atmospheric mercury at urban and rural sites in Poland. For example, Zielonka et al. (2005) registered high emissions of Hg (0.073 kg) from these sources, which resulted in the extremely high value of Hg_p_ dry deposition (43.8 μg m^−2^) during the short-term wintertime measurements (Jan 26–Feb 3, 2004) in Lichwin.

Based on the relationship between Hg_p_ and the wind profile, it was noticed that under less turbulent atmospheric conditions (wind speed 1–2 m/s), the local sources effectively contributed to the increase in concentrations of Hg in aerosol samples. Specifically, meteorological situations associated with high frequency of western, southwestern, and southern advections towards the sampling site in Poznań, mostly in January and February 2014 (65 and 92 % of observations), strongly corresponded to high Hg_p_ concentrations measured in particulate matter samples from these months. In the same period, when northerly and northwesterly winds of medium (1.0–3.0 m/s) or high velocity (>3.0 m/s) occurred (on average 6.6 % of cases), the concentration of Hg in coarse and fine particles declined by ∼10–25 % in relation to samples collected during the W-SW-S advection.

Heterogeneous chemical transformations with other chemical species, i.e., O_3_, SO_2_, CO, PAHs, BTX, radicals, and Pb, were another factor that affected the seasonal variability of particle-bound Hg over the urban site (WIOŚ 2013). During the heating season (Oct–March), high Hg concentrations measured in coarse aerosol samples from Poznań were well correlated with SO_2_ (*R* = 0.80), suggesting the same anthropogenic source of both species, i.e., coal combustion. These observations are in good agreement with data obtained from other urban and traffic sites where elevated values of particle-bound Hg in the ambient atmosphere clearly coincided with high concentrations of other anthropogenic pollutants (Lynam and Keeler 2006; Xu et al. 2015). Moreover, the analysis of concentrations in relation to working/non-working days in Poznań (not shown) revealed that the average values of Hg_p_ concentrations determined in weekday samples were significantly different from those measured in samples collected during weekends (Kruskal-Wallis test, *p* < 0.01).

In contrast to our urban site, the Hg_fine_ concentrations measured in Jeziory, which represents a relatively unpolluted region, did not exhibit a clear seasonal trend (Fig. 2b). Monthly concentrations of fine and coarse particulate Hg were within the ranges of 0.4–3.3 pg m^−3^ and 16.1–38.3 pg m^−3^, respectively. The highest concentration of Hg_coarse_ was measured in January 2014 (non-growing season), mainly as an effect of weather conditions, i.e., low wind speed (<1 m/s) and high emission from regional anthropogenic sources. The monthly minimum of Hg bound to particles was found in August 2014. That decrease in total Hg_p_ was pronounced in the midst of the growing season and was directly associated with much lower emissions from local/regional industrial sources. However, the contribution from other sources, e.g., emission from forest vegetation and soils, was significantly higher. Moreover, in the period from May to September 2014, very unstable atmospheric conditions (winds of 2–10 m/s) and large variability in monthly precipitation (between 13.2 and 84.7 mm) were registered at the forest sampling site. This suggest that high-wind events (>5 m/s) strongly impacted the monthly distribution of coarse and fine Hg at our forest site and caused lower variability of Hg in aerosol samples, probably due to greater dispersion effect or mitigation of locally emitted particles (dry deposition mechanism).

Interestingly, the largest discrepancies between Hg_p_ concentrations measured in fine and coarse particles at the forest site were determined in March 2014, which indicated that the majority of Hg values obtained for Jeziory could presumably be attributed to different sources. These differences between Hg_coarse_ and Hg_fine_ could also be related to small-scale atmospheric processes (e.g., photochemical reactions on aerosol surface) or the increase in air/surface exchange of mercury vapor and other biogenic compounds such as low molecular weight organic acids over forest area. Furthermore, our second station was influenced by relatively low traffic emission (medium-traffic road approx. 4 km from the site) during the whole study period, which could explain low and stable Hg_fine_ concentrations.

### Particulate mercury concentration vs. long-range transport

The spatial Hg_p_ gradient observed in the present study highlighted the long-range atmospheric transport as a significant factor affecting seasonal variability of Hg compounds in the lower troposphere. The use of backward trajectories (BT) from the HYbrid Single-Particle Lagrangian Integrated Trajectory (HYSPLIT) model has become a popular method for interpretation of the results of particulate Hg measurements in relation to regional and global transport of polluted air masses (Li et al. 2008; Cheng et al. 2013; Feddersen et al. 2012; Siudek et al. 2014; Zhang et al. 2015). We examined only Hg_p_ data obtained for the Poznań sampling site (in total 124 BTs) in 2014.

As a result, three different types of air masses were determined and linked to different clusters. The first cluster (N-NW wind sector, 45.2 % of all BTs) represents clean air masses originating from the North Sea and the Atlantic Ocean, and then passing over relatively unpolluted northern European countries. The second cluster comprised of polluted air masses from western and southern European regions, particularly from France, Germany, Austria, Czech Republic, and northern Italy, where intensive industrial/urban activities occurred during the whole study period. In addition, the portion of air masses from Cluster II was associated with local and regional transport from western, southwestern, and southern Poland (Silesia region). These areas were recognized as regions which have a significant contribution to the seasonal pattern of high Hg_p_ observed in Poznań. Trajectories taken into consideration in Cluster III (5.6 % of all BTs simulations) were defined as mixed (sea-land) air masses and they were attributed to the transport over northeastern and eastern European regions.

**Fig. 3:**
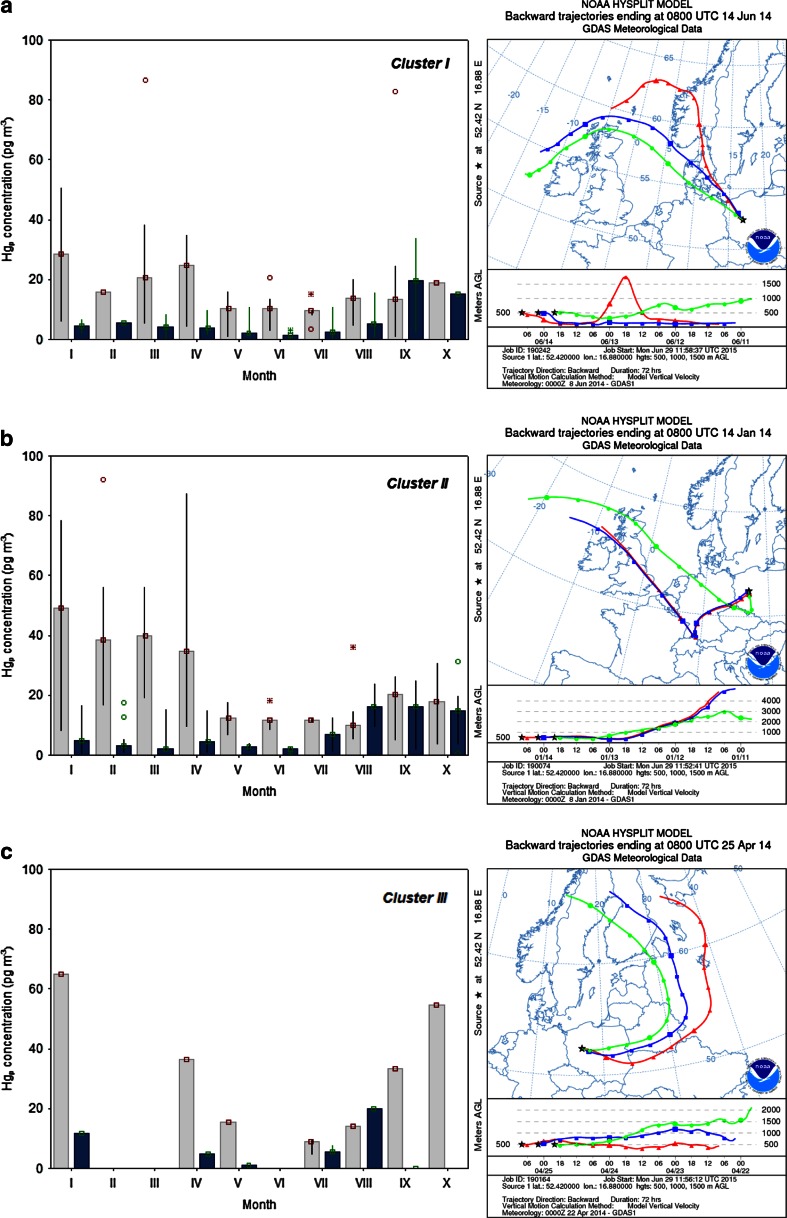
Variation of Hg_p_ concentrations in three clusters of air masses. **a** Cluster I and the 4-day backward trajectories for 16 June 2014. **b** Cluster II and the 4-day backward trajectories computed for Poznań during the episode of high Hg_p_ on 14 January 2014. **c** Cluster III and the 4-day BT simulation for 25 April 2014. *Blue bars* represent Hg_p_ values related to fine aerosol mode, whereas the *gray* ones reflect Hg in coarse particles

Figure 3 illustrates the seasonal variability of particulate Hg concentrations within three clusters reflecting different air masses. The cluster analysis showed relatively various ranges of Hg_p_ concentrations both in fine and coarse particles sampled in Poznań between January and October 2014. The monthly mean concentrations of coarse particulate mercury compared to Hg in fine particles were higher between January and April in all Clusters. At the beginning of the cold season (Jan–March), the elevated Hg_coarse_ concentrations were associated with western, southwestern, and southern air masses (Cluster II), suggesting that the aerosol population over our study domain was under the influence of numerous regional sources from the above sectors. For example, high total particulate mercury concentration (76.5 pg m^−3^) was determined on 14 January 2014—the day when air masses were slowly transported (at relatively low altitude) from western parts of Europe, i.e., Germany, central UK, and northern Czech Republic, including highly polluted areas in southern Poland (Fig. 3b). Siudek et al. (2011) reported very similar episodes of elevated Hg concentrations in size-fractionated airborne particles in Gdynia (northern Poland), and indicated that industrial processes at a regional scale are a key factor. About 20 % of Hg_p_ data taken into account for the cold season (Jan–March) of 2014 in Poznań were additionally attributed to Cluster I, which suggested the significant influence of transboundary transport of Hg species from other anthropogenic sources located in NW-N regions. It should be also noted that due to proximity of the international airport Poznań-Ławica, aircraft emissions could have a significant impact on aerosol loadings and seasonal Hg_p_ transformations in the boundary layer over our urban site. Pirrone et al. (2010) demonstrated a significant role of the civil aviation sector, especially the role of fuel combustion in the global Hg budget; however, they did not estimate its contribution to the total anthropogenic emission of Hg.

In addition, concentrations of coarse particulate mercury were also higher in samples collected in September and October (attributed to Cluster III). This observation suggests that coarse particles can be associated with different mechanical processes, mainly from anthropogenic sources during the heating season. Another possible explanation for seasonal differences in Hg concentration between coarse and fine particles was the impact of ambient temperature and aerosol composition on gas-partitioning of atmospheric mercury. Similar transformations were observed by other authors (Rutter and Schauer 2007; Zhu et al. 2014).

As presented in Fig. 3, Cluster I and II with trajectories computed for the warm season in 2014 (May to August) showed very similar trends (relatively low Hg_coarse_ and Hg_fine_ concentrations which did not exceed 10.0 pg m^−3^). This suggests the substantial decrease of local/regional anthropogenic emission in summer. On the other hand, an increase in fine particulate mercury concentration was observed between July and October, especially in Cluster II and III (Fig. 3). This indicates that such process as the adsorption of gaseous mercury onto submicron aerosols, which were produced by condensation/coagulation of pollutants during local/regional combustion processes, could be an important source of Hg in fine particles.

**Fig. 4:**
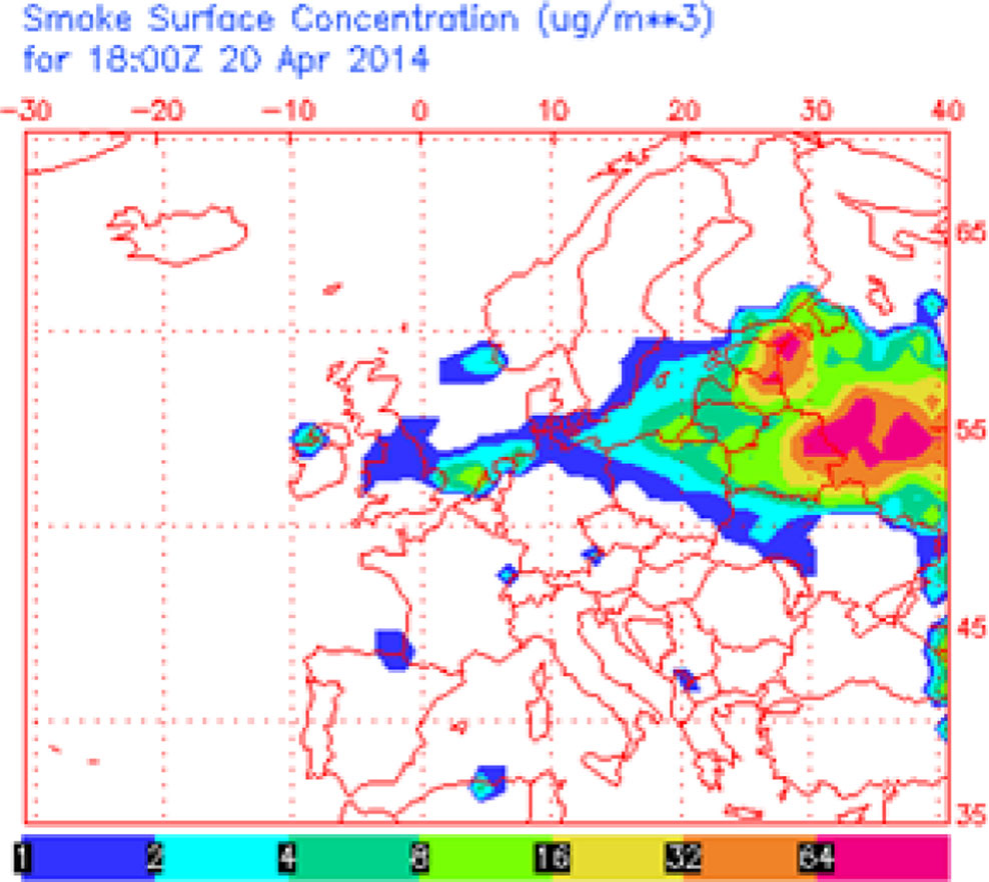
Example of high emission of S compounds from a wildfire episode in Eastern Europe, April 2014 (NAAPS, Navy Aerosol Analysis and Prediction System)

Based on NAAPS model results, it was found that during the measurements in spring (April) and fall (October) of 2014, several large wildfires in Eastern Europe occurred (Fig. 4). It seems that those events could have a potential effect on atmospheric conditions and climate in central Poland. A more recent work by Finley et al. (2009) showed that 15 % of the total mercury released from regional wildfires could be found in particulate-phase Hg and, consequently, its contribution may be considered as a sign of anthropogenic emissions. Thus, the Hg_p_ concentration of 54.7 pg m^−3^ measured on 20 April 2014 in Poznań could be partly attributed to the fire pollution episodes from eastern Ukraine, Russia, and Estonia, as shown in Figs. 3c and 4.

## Summary and conclusions

In the present study, the concentrations of particulate mercury in the atmosphere were investigated using a filter pack method (without a KCl denuder), in the period between April 2013 and October 2014 at two sites in central Poland. The Hg concentrations in particulate matter in Poznań (urban area) were significantly higher than those reported in Jeziory (forest site). The median of PHg in urban fine particles was 4.0 ± 9.1 pg m^−3^, whereas Hg in coarse aerosols was 16.4 ± 45.3 pg m^−3^. Higher values of coarse Hg concentrations were observed during the episodes of winter pollution as compared to summer or spring measurements. The similar trend was found for Hg associated with fine aerosol fraction; however, the concentration range and variation were relatively lower. The seasonal variations of Hg in both the fractions at the urban site were higher than in the woodland protected area, which indicated a large influence of local anthropogenic sources. In this study, several factors, including air temperature, wind speed and direction, photochemistry, and precipitation amount, were identified as crucial for atmospheric transformations of Hg in particulate phase. During cold periods, low air temperature correlated well with elevated Hg_coarse_ concentrations, suggesting the predominance of gas-to-particle partitioning in the atmosphere. The main sources of Hg were determined based on meteorological analyses and backway trajectories. It was found that locally and regionally emitted Hg species significantly affected aerosol chemistry and properties within the examined urban-forest transect.
